# Physical Fitness and Academic Performance in Normal Weight, Overweight, and Obese Schoolchild Handball Players in Qatar: A Pilot Study

**DOI:** 10.3389/fpsyg.2020.616671

**Published:** 2021-01-13

**Authors:** Souhail Hermassi, Lawrence D. Hayes, Nicola Luigi Bragazzi, René Schwesig

**Affiliations:** ^1^Sport Science Program, College of Arts and Sciences, Qatar University, Doha, Qatar; ^2^School of Health and Life Sciences, University of the West of Scotland, Glasgow, United Kingdom; ^3^Postgraduate School of Public Health, Department of Health Sciences (DISSAL), Genoa, Italy; ^4^Department of Orthopaedic and Trauma Surgery, Martin-Luther-University Halle-Wittenberg, Halle, Germany

**Keywords:** schoolchild, anthropometrics, achievement, youth players, academic performance

## Abstract

This study aimed to investigate the relationships between physical fitness and academic performance in youth handball players of different BMI classifications. Thirty-three male handball players (age: 10.3 ± 0.61 years; body mass: 47.1 ± 12.1 kg; height: 1.43 ± 0.09 m; BMI: 23.1 ± 4.37 kg/m^2^; body fat: 20.6 ± 6.27%) were recruited from the Qatar handball first league and were assigned to their BMI age-adjusted groups (i.e., normal weight, overweight, and obese). Measurements included anthropometric data (height, mass, body mass index (BMI) and body fat percentage (%BF), and physical performance tests: agility T-half test; squat jump (SJ), and countermovement jump (CMJ), 10 and 15 m sprint; medicine ball throw (MBT). Aerobic capacity was evaluated using the Yo-Yo Intermittent Recovery Test level 1 (Yo-Yo IR1). Academic achievement was assessed through school records of grades point average (GPA) of Mathematics, Science and Arabic. None academic performance parameter and four physical performance parameters (agility T-half: *p* = 0.035; CMJ: *p* = 0.001; SJ: *p* = 0.007; sprint 10 m: *p* = 0.028) were different between BMI related groups. In 43% (3/7) of performance parameters and all academic parameters, the normal weight group showed the highest performance level, whereas the overweight group had the best performance in both sprint tests. The obese group was only superior in the medicine ball throw, but not at the *p* < 0.05 level. A relevant relationship (*r* > 0.5) between academic and physical performance parameters was only found between Yo-Yo IR 1 and science (*r* = 0.548). A relevant correlation were found between CMJ and BMI (*r* = −0.569). The agility T-half test was correlated with CMJ (*r* = −0.614) and 10 m sprint (*r* = 0.523). These findings suggest being overweight or obese are related to science academic performance among schoolchildren athletes in Qatar. Possibly, a normal BMI could positively influence academic performance. Physical education teachers, staff, and administrators should be cognizant that health promotion interventions improving composition may have the additional potential to improve dimensions of academic performance.

## Introduction

Concerns for physical health, physical fitness, wellbeing, and academic achievement of children are at unprecedented levels. Childhood obesity has risen, although currently stabilizing, and childhood fitness has declined (Hruby and Hu, 2015; Sahoo et al., 2015). Academic achievement is declining concomitantly with these decreases in health (El Ansari and Stock, 2010; Bass et al., 2013). However, these parameters are often treated disparately. It is recognized that obesity is associated with adverse neurocognitive outcomes (Anderson et al., 2019). The relationship between weight, obesity, and brain function relates to executive function or self-regulatory cognitive processes associated with monitoring of thought and goal-related behaviors (Reinert et al., 2013). These effects appear to be modifiable, and a systematic review and meta-analysis of 20 studies found weight loss was associated with improvements in performance across various cognitive domains (Veronese et al., 2017).

Excess body fatness, typically determined quickly and easily with BMI is a major public health challenge worldwide (Malecka-Tendera and Mazur, 2006). Being overweight (BMI: 25–29 kg/m^2^) or obese (BMI: >30 kg/m^2^) accompanies poorer academic performance (Davis and Cooper, 2011), while high levels of physical activity and fitness and low body fat are associated with superior cognition (Ruiz et al., 2010). Some studies have suggested obese children have lower intelligence quotient (IQ) and exhibit worse executive function, attention, memory, and motor skills compared with normal weight contemporaries (Ruiz et al., 2010).

Promising associations between physical activity levels, motor competence and fitness, and academic performance have been reported by observational cohort studies and small controlled laboratory studies (Singh et al., 2012; Donnelly et al., 2016; Fernandes et al., 2018), so it is plausible that school-based health interventions during physical education lessons could impact students’ academic performance. However, conflicting results have emanated from large school-based intervention studies (Ericsson and Karlsson, 2014; Kall et al., 2014; Bugge et al., 2018).

Physical fitness is putatively a powerful indicator of child and adolescent health (Ortega et al., 2008). The primary health-related components are cardiorespiratory fitness, muscular strength, and speed-agility (Ortega et al., 2008). The relationship between cardiorespiratory fitness and academic success has been the primary focus of research to date, concluding higher cardiorespiratory fitness is associated with superior academic achievement in children (Esteban-Cornejo et al., 2014; Donnelly et al., 2016; Aadland et al., 2017). However, far less investigated is the relationship between other physical traits such as strength, power, and agility, and academic performance (Donnelly et al., 2016). In this regard, Kao et al. (2017) emphasized the importance of research concerning muscular fitness and its association with cognitive health.

Recent research has begun to establish an association between physical and cognitive variables, demonstrating that physical activity (PA) is not merely coincidentally related to cognitive function (Donnelly et al., 2016; Fernandes et al., 2018; Cadenas-Sanchez et al., 2020; Gil-Espinosa et al., 2020). In addition to the direct role exercise plays in brain plasticity, PA is important for cognitive development, mood state, learning, memory, and concentration (Aberg et al., 2009; Donnelly et al., 2016; Fernandes et al., 2018). The association between physical fitness and academic attainment (Chomitz et al., 2009; Kwak et al., 2009; Wittberg et al., 2012) promotes the justification for increased PA in children and adolescents for the reversal of current disease trends and for the improvement of academic achievement. Of the few studies that have concerned PA and academic achievement, few have examined the relationship between obesity, standardized fitness assessments, and academic achievement (Chomitz et al., 2009; Davis and Cooper, 2011; Cadenas-Sanchez et al., 2020). Therefore, research is needed to examine each component of physical fitness and its relationship with academic achievement in schoolchildren within one investigation. Likewise, it is necessary to differentiate fitness and body-fatness indicators and academic performance in schoolchildren. This research area scarcely concerns countries from the Gulf region, and this geographical area has unique features which means research from other parts of the world may not be applicable to the Gulf. Therefore, this study investigated school performance (e.g., Arabic, mathematics and science), and physical fitness of normal, overweight, and obese child handball athletes in Qatar. The aims were (a) to describe physical and academic performance stratifying based on BMI and (b) to evaluate interactions between BMI, physical fitness, and academic performance.

## Materials and Methods

### Participants

The investigation was completed during the in-season period, from January to February 2020. Thirty-three male handball players were recruited from the Qatar handball first league (age: 10.3 ± 0.6 years; body mass: 47.1 ± 12.1 kg; height: 1.43 ± 0.09 m; BMI: 23.1 ± 4.4 kg/m^2^; body fat: 20.6 ± 6.3%). Participants trained ubiquitously, supervised by the two of the team’s coaches, from the commencement of the competitive season (September) until the completion of the current study (March). Players were divided into three groups depending on BMI age-adjusted values (Harrington et al., 2013): normal weight (*n* = 11; BMI: 18.0–20.9 kg/m^2^); overweight (*n* = 11; BMI: 21.0–22.9 kg/m^2^); obese (*n* = 11, BMI: >23.0 kg/m^2^). Six players were left-handed and 27 players were right handed. Players trained on average 3.2 ± 0.5 h/week, and training otherwise consisted of motor skills (60% of session time) and basic team handball techniques through playing games (40% of session time). In addition, subjects participated in physical education lessons at school, which lasted for 40 min and consisted mainly of ball games.

Participants did not report any musculoskeletal injuries in the 4 weeks prior to the commencement of the study and did not train between the pre-test and 24 h after performing the test procedures. Prior to the commencement of the present study, all players and parents were advised about the potential risks and benefits associated with participation in this study. All players and/or their parents/guardians provided a signed, written, informed consent. Participants were fully familiarized with procedures and were informed about the possibility of withdrawing from the study at any time without repercussion. The project was conducted in accordance with declaration of Helsinki and its guidelines and the university’s institutional review board (QU-IRB 1163-EA/19) approved the study’s protocol.

#### Procedures and Evaluations

Testing was completed on an indoor handball court, at the same time of day (from 18:00 h–20:00 h), under similar environmental conditions (temperature 20.5 ± 0.5°C, relative humidity 60 ± 5%). Participants were asked to maintain their normal consumption habits of food and fluids. However, they abstained from performing any vigorous physical activity, drinking caffeine-containing beverages, and eating, for 24, 4, and for 2 h before testing, respectively. Tests were performed over a period of 4 days in a fixed order to elicit similar fatigue effects between players. On the first day, anthropometric measurements preceded vertical jump tests (SJ and CMJ). Sprint performance was determined on day two, and on the third day, the agility T-Half test and medicine ball throw were conducted, followed by the Yo-Yo IR1 on the fourth day.

Two weeks after the initial testing period, fitness tests from day 1 to day 3 were repeated (i.e., SJ, CMJ, sprints, T-Half agility, medicine ball throw) to allow for assessment of test-retest reliability. Scores of the second set of tests were considered for analyses. Anthropometric assessments and the Yo-Yo IR1 test were only done once at the initial testing period for convenience reasons related to time and players’ schedules.

### Day 1

#### Anthropometry

Anthropometric measurements comprised standing height (Holtain stadiometer, Crosswell, Crymych, Pembrokeshire, United Kingdom) and body mass (model TBF 105; Tanita Corporation of America, Inc., Arlington Heights, IL) and were measured to the nearest 0.1 cm and 0.1 kg, respectively. Body mass index (BMI) was calculated as the ratio between body mass (kg) and body height squared (m^2^). Percent body fat (%BF) was assessed using the skinfold method with a Harpenden caliper (Baty International, Burgess Hill, Sussex, United Kingdom). Standardized procedures were followed as per the guidelines of International Society for the Advancement of Kinanthropometry (ISAK). %BF was predicted from the sum of four skinfolds biceps, triceps, subscapular, and suprailiac), using the Womersley and Durnin equation (Womersley and Durnin, 1973). Participants were then assigned to their BMI age-adjusted percentile groups (i.e., normal weight, overweight, and obese).

#### Squat (SJ) and Counter Movement Jump (CMJ) Tests

Before commending the jump tests, participants completed a general warm-up procedure that included 5 min of running, stretching of lower limbs muscles, and 2 min of jumping exercises. SJ and CMJ heights were assessed using the Optojump photoelectronic cells (Optojump Next, Microgate, Italy) (Glatthorn et al., 2011). Jump heights were determined from the recorded contact and flight time of jumps with a sampling rate of 1 kHz. The SJ began at a 90° knee angle; avoiding downward movement, participants performed a vertical jump by pushing their body upward with the legs. The CMJ began from an upright position, whereby participants made a rapid downward movement to a knee angle of approximately 90°, arms akimbo and simultaneously beginning to push-off, after being instructed to jump as fast and high as possible. Both tests were performed without an arm swing by fixing hands at the level of the pelvis and with knees and ankles extended at take-off and landing. The highest of four jumps was recorded for each test, and a 30 s recovery was given between each jump.

### Day 2

#### Sprint Tests

Participants performed a 15 min warm up, consisting of 10 min running, change of direction activities, and dynamic stretching. Subsequently, participants sprinted 15 m from a standing position 0.2 m behind the first photocell beam 15 and 30 m sprint times were recorded by paired photocells (Racetime 2 SF, Microgate, Italy) located 1 m above the ground at the start and finish. Three trials were interspersed by 6–8 min of recovery, and the fastest trial was retained for further analyses.

### Day 3

#### Change of Direction (T-Half Test)

A 10 min warm-up including jogging, lateral displacements, dynamic stretching, and jumping was conducted prior to each test. T-Half tests (Sassi et al., 2009) data were recorded using electronic timing sensors (photocells, Kit Racetime 2 SF, Microgate, Italy) set at 0.75 m above the floor, 3 m apart and facing each other at the starting line A. Each trial began with the front foot 0.2 m behind line A. Subsequently, participants sprinted forward to cone B and touched its base with their right hands. Facing forward and without crossing their feet, they shuffled to the left to cone C and touched its base with their left hand. Then participants shuffled right to cone D and touched its base with their right hand, then ran back to the left to cone B and touched its base. Finally, they ran backward as quickly as possible, returning to line A. Participants who crossed one foot in front of the other, failed to touch the base of a cone, and/or failed to face forward throughout had to repeat the trial. Participants repeated the test until two successful trials were done, with 3 min of rest between trials, and only the best trial was considered in the analyses.

#### Medicine Ball Throw

Prior to the overhead medicine ball throw test, each subject performed a 5 min warm up, consisting of 3 min running and dynamic activities. Throws were performed using 21.5 cm diameter rubber medicine balls weighing 2 kg. A brief description of the optimal technique was given, identifying an optimal release angle to achieve a maximum distance (Negrete et al., 2010). The sitting player grasped the medicine ball with both hands, and on the given signal forcefully pushed the ball from the chest. The score was measured from the front of the sitting line to the place where the ball landed. Three trials were performed, separated by 1 min of rest, and the best result was recorded to 0.01 m.

### Day 4

#### The Yo-Yo Intermittent Recovery Test Level 1 (Yo-Yo IR1)

The Yo-Yo IR1 was conducted as described by Krustrup et al. (2003). The reliability of the test has been established with a coefficient of variation of 3.6% and an intraclass correlation (ICC) coefficient of 0.94 (Krustrup et al., 2003). A standardized warm up comprised 5 min of low-intensity running. Then, participants completed 20 m shuttle runs at increasing velocities until exhaustion, with 10 s of active recovery (2 m × 5 m of jogging) between each 20 m. If participants failed twice to reach the line in time (objective criterion) and/or felt unable to complete another shuttle at the required speed (subjective criterion), their test was terminated. The total distance covered was considered as the test score.

#### Academic Performance

School records were used to retrospectively determine academic performance. To evaluate the players’ academic performance, the students’ actual Grade Point Average (GPA) along with their subject-specific percentage in Arabic language, Mathematics, and Science were obtained at the first semester of the academic year 2019–2020 from the school records.

#### Statistical Analysis

Statistical analysis was performed using SPSS version 25.0 for Windows (SPSS Inc., IBM, Armonk, NY, United States). Data were tested for normal distribution (Shapiro-Wilk Test) and homogeneity of variance (Levene-Test for equality of variances). Mean differences of anthropometric and performance parameters between groups (normal weight vs. overweight vs. obese) were tested using a one-way analysis of variance (ANOVA) (Bortz, 1999). Differences between means were considered meaningful if *p* < 0.05 and partial eta-squared (η_*p*_^2^) > 0.15 (Richardson, 2011). Alpha level is reported as exact *p* values and was not set dichotomously as significant or non-significant as recommended by Hurlbert et al. (2019). *Post hoc* pairwise comparisons were conducted using Bonferroni corrected T-tests. Due to the relatively small number of cases in each age group (*n* = 11) and in order to avoid an overestimation of mean differences, interpretation of results was primarily based on η_*p*_^2^. For clarity, magnitude of group differences were interpreted using the following criteria: 0.02, a small difference; 0.13, a moderate difference; 0.26, a large difference. The effect size (d) (the mean difference of scores divided by the pooled SD) was calculated for each parameter (Hartmann et al., 1992), as is interpreted as trivial (<0.20), small (≥0.20–0.49), moderate (≥0.50–0.79), and large (≥0.80). Pearson’s product moment correlations were calculated and used to determine the relationship between anthropometric and performance (physical and academic) parameters. A magnitude of correlation (*r*) between the measures of <0.1, 0.1–0.3, 0.3–0.5, 0.5–0.7, and 0.7–0.9, >0.9, were considered trivial, small, moderate, large, very large, and almost perfect, respectively.

## Results

### Normal Distribution and Variance Homogeneity

The variables age (*p* = 0.161), medicine ball throw (*p* = 0.493), T-half test (*p* = 0.306), CMJ (*p* = 0.226) and 10 m sprint (*p* = 0.625) were normally distributed. Regarding variance homogeneity, four parameters (weight: *p* < 0.001; BMI: *p* < 0.001; Arabic: *p* = 0.012, science: *p* < 0.001) were not homogenous in variance. Otherwise, all *p*-values were above 0.065 (SJ) indicating that the variances from all other variables were not different.

### Anthropometric Data

Naturally, body mass (*p* < 0.001), BMI (*p* < 0.001), and%BF (*p* = 0.002) were different between groups at the *p* < 0.05 level (Table 1). The largest difference was found for BMI (η_*p*_^2^ = 0.719). Age (*p* = 0.588) and height (*p* = 0.997) were not different between groups.

**TABLE 1:** Comparison of anthropometric parameters between three groups.

	normal weight (*n* = 11)	overweight (*n* = 11)	obese (*n* = 11)	Variance analysis/effects *p* (η_*p*_^2^)	Observed power	Significant partial effects *p* (d)
Age (years)	9.690.70	9.380.94	9.700.96	0.588 (0.033)	0.131	–
Height (m)	1.420.08	1.420.11	1.420.10	0.997 (0.000)	0.050	–
Weight (kg)	38.94.11	45.34.42	57.415.3	**<0.001 (0.415)**	**0.988**	normal weight vs. obese: <0.001 (1.91) overweight vs. obese: 0.010 (1.23)
BMI (kg/m^2^)	19.20.68	22.20.49	28.04.05	**<0.001 (0.719)**	**1.000**	normal weight vs. overweight: 0.009 (5.13) normal weight vs. obese: <0.001 (3.72) overweight vs. obese:<0.0101 (2.56)
Body fat (%)	16.74.37	20.55.73	25.65.51	**0.002 (0.328)**	**0.931**	normal weight vs. obese: 0.001 (1.80)

*Values are given as mean ± SD. Meaningful effects (main effect criteria: *p* < 0.05 and η_*p*_^2^ > 0.15 and observed power >0.60) are highlighted in bold.*

### Physical Performance Data

With the exception of medicine ball throw (obese: 4.08 ± 1.05 m) and sprinting parameters (overweight: 10 m: 2.43 ± 0.35 s, 15 m: 3.60 ± 0.46 s), athletes from the normal weight group showed the highest performance level in all parameters (Table 2).

**TABLE 2:** Comparison of performance parameters between three groups.

	**Normal weight (*n* = 11)**	**Overweight (*n* = 11)**	**Obese (*n* = 11)**	**Variance analysis/effects *p* (η_*p*_^2^)**	**Observed power**	**Significant partial effects p (d)**
**Physical performance parameters**						
Yo-Yo IR 1 (m)	902227	695274	782320	0.205 (0.094)	0.325	–
Medicine ball throw (m)	3.940.91	3.770.48	4.081.05	0.659 (0.026)	0.113	–
Agility T-half (s)	8.681.33	9.791.33	10.21.43	**0.035 (0.190)**	**0.643**	normal weight vs. obese: 0.038 (1.10)
CMJ (cm)	17.52.69	13.52.92	12.82.76	**0.001 (0.363)**	**0.962**	normal weight vs. overweight: 0.005 (1.43) normal weight vs. obese: 0.001 (1.73)
SJ (cm)	17.23.40	13.23.19	13.62.26	**0.007 (0.269)**	**0.843**	normal weight vs. overweight: 0.009 (1.21) normal weight vs. obese: 0.030 (1,27)
Sprint 10 m (s)	2.680.48	2.430.35	2.960.55	**0.028 (0.200)**	**0.675**	overweight vs. obese: 0.024 (1.18)
Sprint 15 m (s)	3.730.51	3.600.46	4.120.72	0.083 (0.144)	0.495	–
**Academic performance parameters**						
Arabic	88.18.33	80.915.2	83.517.7	0.470 (0.046)	0.170	**–**
Mathematics	91.415.0	76.714.4	87.516.8	0.064 (0.158)	0.541	**–**
Science	93.56.23	83.17.70	85.515.6	0.057 (0.164)	0.562	**–**

*Values are given as mean ± SD. Meaningful effects (main effect criteria: *p* < 0.05 and η_*p*_^2^ > 0.15 and observed power >0.60) are highlighted in bold.*

Between group differences existed for the T-Half Test (*p* = 0.035, η_*p*_^2^ = 0.190), CMJ (*p* = 0.001, η_*p*_^2^ = 0.363) and SJ (*p* = 0.007, η_*p*_^2^ = 0.269). In all parameters, partial group effects were calculated for normal weight vs. obese. For CMJ and SJ, the comparison between overweight and normal weight also yielded a difference (CMJ: *p* = 0.005; SJ: *p* = 0.009). In two parameters (Yo-Yo IR1, medicine ball throw), the obese group showed a higher performance than the overweight group, but not at the *p* < 0.05 level.

### Academic Performance Data

The academic parameters (Table 2) generated the largest difference between groups for science (*p* = 0.057; η_*p*_^2^ = 0.164). The magnitude difference for mathematics was comparable, but did not reach the *p* < 0.05 level (*p* = 0.064; η_*p*_^2^ = 0.158). For all parameters studied, the normal weight group had the highest performance level. In contrast, the overweight group showed the lowest level in all academic performance parameters. However, no differences at the *p* < 0.05 level were detected.

**FIGURE 1:**
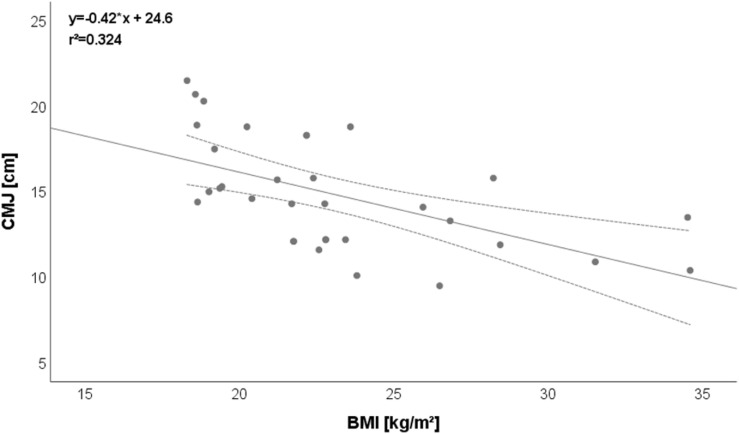
Relationship between BMI and CMJ. Please note that one dot can represent several subjects.

**FIGURE 2:**
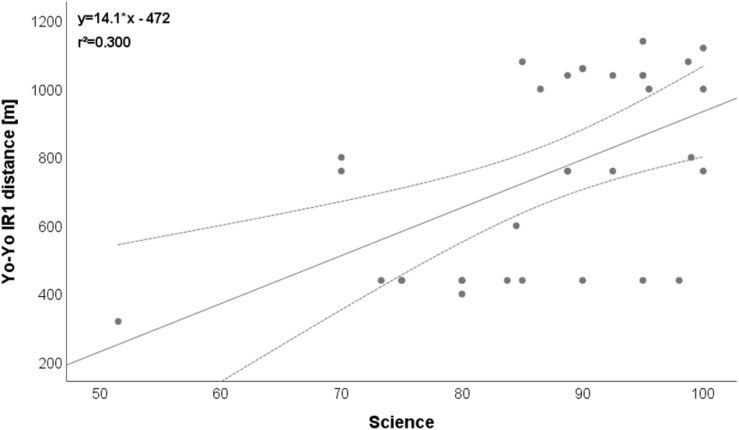
Relationship between Yo-Yo IR1 and science. Please note that one dot can represent several subjects.

Regarding relationships between different categories of parameters (anthropometric vs. physical performance vs. academic performance), two relevant (*r* > 0.5) correlations were detected: BMI vs. CMJ: *r* = −0.569 (Figure 1), Yo-Yo IR1 vs. science: *r* = 0.548 (Figure 2).

## Discussion

This study examined anthropometry, physical fitness, and academic performance in normal weight, overweight, and obese schoolchild handball players. The main finding of this investigation was athletes from the normal weight group had the highest performance in all physical fitness tests, with exception of medicine ball throw and sprinting. Only some physical performance parameters (CMJ, SJ, sprint 10 m, Agility T-half) were different between several BMI groups. However, for all academic areas, the normal weight group exhibited the highest group mean. In contrast, the overweight group had the lowest mean value in all academic areas. Therefore, it seems, that a high cardiorespiratory fitness is positive associated with a higher level of academic performance, but more evidence is necessary to confirm this relationship. In contrast, anaerobic performance tests (e.g., sprinting, jumping, throwing) were not related to academic performance at ages studied herein.

### Physical Performance Data

Sprinting for short distances and the ability to accelerate and decelerate with and without change of directions are fundamental in team-handball (Wagner et al., 2014, 2017; Hermassi et al., 2019b,c). Normal weight players showed the highest performance level in jumping, but not for the medicine ball throw and sprint tests. The difference in sprint times (10 m and 15 m) between groups ranged from 11 to 17%. In many game situations, such as during a fast break or while returning to defense after a ball loss, players sprint for about 10–30 m (Hermassi et al., 2019c).

Concerning throwing performance, the mean difference between groups was 18, 9, and 7% between normal weight and obese, normal weight and overweight, and overweight and obese respectively. Because strength and power are thought to be important for throwing velocity, the medicine ball throw is a valid test to predict power of upper limb in handball players (Hermassi et al., 2010, 2019a). Higher values of maximum power and muscle strength of the upper limbs provide an advantage for the obese group in this regard. However, whether increased strength and power outweighs the disadvantages experienced in other physical domains such as jumping, agility, and sprinting is improbable, and is likely an artifact of increased lean mass and adipose mass physical tests mentioned, such as the SJ and CMJ.

For the SJ and CMJ, pairwise differences between normal weight and obese existed at the *p* < 0.05 level. Likewise, comparison between overweight and normal weight yielded a difference. The average difference between BMI groups ranged from 27% (CMJ) to 21% (SJ). As handball is a contact sport, in which jumping, hitting, blocking, and pushing are common, strength is necessary to compete at a high level in youth and adult handball (Hammami et al., 2019; Hermassi et al., 2019a). These effects likely occurred as a result of having to accelerate less mass against gravity, and we propose that relative power (i.e., power/weight) is more important in vertical jumps than horizontal sprints, and this is why the overweight group could show the highest level of sprint performance (10 and 15 m). However, only for the parameter sprint 10 m the performance difference between overweight and obese group was at the *p* < 0.05 level.

Agility is a key determinant of optimal performance in many invasion sports and one of the most important factors among youth handball players (Hermassi et al., 2019a,b). In our study, performance differences between groups were different in the T-Half Test. However, the peak rate of agility development occurs at approximately 13–14 years in males (Vänttinen et al., 2011), so between group differences may become more pronounced at that age.

Repeatedly performing high-intensity actions are a requirement in handball. Difference in Yo-Yo IR 1 performance between BMI groups was 27% between normal weight and overweight, 23% between normal weight and obese, and 5% between overweight and obese. Aerobic fitness is an important element in handball (Hammami et al., 2019). Although differences in Yo-Yo IR1 performance did not reach the *p* < 0.05 or η_*p*_^2^ > 0.15 level, we propose a 23 or 27% difference in Yo-Yo IR 1 performance is of a considerable magnitude to exert an effect in a game situation.

### Academic Performance

One meaningful group effect from the ANOVAs was observed for science. In all academic parameters, the normal weight group had the highest mean performance and the obese group the lowest, but not at the *p* < 0.05 level. A substantial body of research has convincingly demonstrated obesity and poorer academic performance are associated, commencing as early as kindergarten (Wu et al., 2017). In this context, obese children perform worse in tests than slimmer counterparts, and are consequently more likely to be held back a grade and are less likely to attend university. While a substantial amount of research focusses on the extent to which educational performance predicts later-life body mass, a growing body of literature focuses on the reverse causative relationship, concerning how body mass affects educational outcomes (Ding et al., 2009). Results suggest that while obesity is not associated with lower test scores (Datar and Sturm, 2006), it does appear to predict lower GPA (Li et al., 2008). The question of why obesity would be associated with GPA but not with test scores remains unknown, and is likely multifactorial, subject to varying social constraints at the intersection of race, sex, and body size in the classroom.

Obesity has a known mechanistic influence on cognition, learning, and memory (Li et al., 2008; Yu et al., 2009). However, weight−related bias or discrimination may influence self−esteem and behavioral problems, thus mediating or moderating children’s academic performance (Fowler-Brown et al., 2010; Griffiths et al., 2010). It is still unclear whether excess adiposity itself affects academic performance, or if this effect is mediated by other factors observed in individuals with obesity.

Aerobic fitness is known to predict academic performance in large-scale cohorts from elementary school up to secondary school (Castelli et al., 2007; Chen et al., 2013; Donnelly et al., 2013; Rauner et al., 2013; Torrijos-Nino et al., 2014), adding credence to the association during early life. Exercise and physical activity directly improve aerobic capacity, which directly affects brain plasticity (Hillman et al., 2008), and is associated with cognitive health, better cognitive abilities, larger brain structures (Esteban-Cornejo et al., 2014), elevated brain function (Esteban-Cornejo et al., 2014), and improved memory (Hillman et al., 2005), along with neurocognitive functions and cognitive control (Hillman et al., 2005).

### Limitations of the Study

The main limitation of the present study is its cross-sectional nature and that sexual maturation was not considered. A previous study indicated that maturation status explains a small amount of variance in aerobic fitness independent of%BF (Mota et al., 2002). Future studies should include an assessment of sexual maturation when examining the relationship between anthropometry and physical performance in youths. Since the design of this study was cross-sectional, it is hard to investigate the causality in the studied relation. The main methodological limitations of this study were a small sample size (11 subjects in each group) and the absence of a non-athletic control group. Furthermore, it is counterintuitive and surprising that 2/3 of participants with high physical activity levels (∼6 h training per week and physical education classes) showed a body mass above that recommended for their age. Therefore, future studies should also collect data regarding nutrition practices of the handball players (e.g., food diaries). Maybe, this could be a selection effect and prevents the generalization regarding the general population. Further profiling studies of elite youth male and female handball players are necessary in order to establish reference data.

## Conclusion

The present study compared physical fitness and academic performance, as a factor of anthropometry in handball playing children. Throwing performance and aerobic capacity were not different between BMI groups. Obese children had superior medicine ball throw performance whilst the normal weight group exhibited the best jumping and agility performance. These data can be used to generate body-weight specific reference data for schoolchild handball players, coaches, and trainers. Future studies with larger sample sizes and additional parameters (e.g., lean mass) are required for confirmation of our preliminary observations.

## Data Availability Statement

The raw data supporting the conclusions of this article will be made available by the authors, without undue reservation.

## Ethics Statement

The studies involving human participants were reviewed and approved by IRB| Qatar University. Written informed consent to participate in this study was provided by the participants’ legal guardian/next of kin.

## Author Contributions

SH and NB: conceptualization, supervision, and writing – original draft. SH and RS: formal analysis, investigation, and methodology. SH: project administration: SH and LH: writing – review and editing. All authors contributed to the article and approved the submitted version.

## Conflict of Interest

The authors declare that the research was conducted in the absence of any commercial or financial relationships that could be construed as a potential conflict of interest.
